# Concerted Flexibility of Chromatin Structure, Methylome, and Histone Modifications along with Plant Stress Responses

**DOI:** 10.3390/biology6010003

**Published:** 2017-01-16

**Authors:** Ana Paula Santos, Liliana J. Ferreira, M. Margarida Oliveira

**Affiliations:** Instituto de Tecnologia Química e Biológica António Xavier, Universidade Nova de Lisboa, Genomics of Plant Stress Unit. Av. da República, 2780-157 Oeiras, Portugal; lferreira@itqb.unl.pt (L.J.F.); mmolive@itqb.unl.pt (M.M.O.)

**Keywords:** chromatin structure, epigenetics, DNA methylation, histone modifications, epi-drugs, epi-mutations

## Abstract

The spatial organization of chromosome structure within the interphase nucleus, as well as the patterns of methylome and histone modifications, represent intersecting layers that influence genome accessibility and function. This review is focused on the plastic nature of chromatin structure and epigenetic marks in association to stress situations. The use of chemical compounds (epigenetic drugs) or T-DNA-mediated mutagenesis affecting epigenetic regulators (epi-mutants) are discussed as being important tools for studying the impact of deregulated epigenetic backgrounds on gene function and phenotype. The inheritability of epigenetic marks and chromatin configurations along successive generations are interpreted as a way for plants to “communicate” past experiences of stress sensing. A mechanistic understanding of chromatin and epigenetics plasticity in plant response to stress, including tissue- and genotype-specific epigenetic patterns, may help to reveal the epigenetics contributions for genome and phenotype regulation.

## 1. Introduction: The Structural Chromatin Organization

In eukaryotes, the genome size is highly variable between organisms [1]. There is a remarkable difference between the linear length of DNA and the size of the nucleus and, thus, the entire DNA molecule has to be efficiently compacted in order to fit inside the physically restricted space that is the three-dimensional nucleus. Different levels of chromatin organization are achieved by the association of DNA with structural proteins, such as the core and linker histone proteins. At the basic level of chromosome structure, the DNA is bound to histone proteins forming the nucleosomes which represent the basic element of chromatin. Mitotic chromosomes possess high levels of chromatin compaction implying the acquisition of additional levels of chromatin organization, defined as the large-scale level of chromatin organization [2]. From cytogenetics analysis, chromatin can be defined as euchromatin or heterochromatin depending on the compaction state [3,4]. Heterochromatin is highly condensed and rich in repetitive sequences, with the more compact state negatively correlated with the transcriptional competence. In contrast, euchromatin is typically characterized as having a more relaxed structure, with this state being associated with nucleosomes that more spaced apart and irregularly arranged, ensuring transcriptional competence [5].

### Methods to Evaluate Chromatin Organization

The way interphase chromatin is organized in the three-dimensional space is crucial in genome regulation. Understanding the arrangement of chromatin in the interphase nucleus has benefited from great improvements in fluorescence in situ hybridization (FISH) with chromosome-specific probes, together with crucial improvements in microscope technology. These advances, initially applied in animal cells, showed that chromosomes occupy distinct territory domains in the interphase nucleus [6,7]. In plants, the investigation of chromosomal structure in interphase nuclei has not been straightforward because of the high level of dispersed repetitive sequences, making it difficult to generate strong and specific chromosome probes [8,9]. The visualization of chromosome territories in interphase nuclei was first achieved in interspecific and intergeneric hybrid plants by using genomic in situ hybridization (GISH) in meiotic cells to discriminate genomic origins [10]. The way plant chromosomes are organized within interphase nuclei was analysed in distinct cell types and in various plant species and these studies showed a high diversity of interphase chromosome arrangements [10,11,12,13]. In wheat/rye addition and/or translocation lines, the chromosomes appeared as elongated domains, stretched across the diameter of the interphase nucleus, with both arms physically adjacent and with centromeres and telomeres located at opposite extremes of the interphase nucleus, displaying a typical Rabl configuration [12,13,14]. This typical Rabl arrangement of chromosomes is suggested to involve specific attachment of telomeres and centromeres to some component(s) of the nuclear envelope. In rice, so far, a “real” painting of chromosomes in interphase nuclei has not yet been achieved. However, FISH with probes made of bacterial artificial chromosomes (BAC) clones containing fractions of genomic DNA spanning chromosome 1, or with centromeric, telomeric, and ribosomal probes, has increased our knowledge of chromosome organization in rice interphase nuclei [13]. In diploid rice, telomeres and centromeres are dispersed around the nuclear periphery in meristematic cells from the root-tip and, thus, not presenting a classic Rabl configuration [15]. Nevertheless, the Rabl arrangement of chromosomes was found in other cell types of rice root sections, namely in larger cells, presumably due to endoreduplication, which will originate the xylem [15]. *Arabidopsis thaliana* and its close relative *A. lyrata* are, so far, the only plants where a “real” chromosome painting within interphase nuclei has been obtained, and that was made possible by using a series of BACs as DNA probes [16]. These analyses enabled the visualization of telomeres clustered and located near the nucleolus, and also the centromeres (chromocenters) positioned at the nuclear periphery from where chromatin loops (0.2 to 2 Mb in length) emanate forming a “rosette-like” structure of chromosome territories [16]. These studies regarding the organization of chromosome territories, genes, and intracellular RNA dynamics have been based on cytogenetics conducted mainly in fixed cells through in situ hybridization (ISH) which, although still a powerful tool to study nuclear architecture, only provides restricted information on existing structures at the time of fixation. Thus, there is a growing interest in live-cell imaging techniques and one possible strategy to follow the dynamics of intracellular RNA and proteins in living cells may involve fluorescent labelling [17,18]. Studies on living plant cells can benefit from the use of an optical imaging chamber that enables visualization for long periods [19]. The fluorescence recovery after photobleaching (FRAP) methodology allows the analysis of the molecular dynamics in living cells, including histones and other chromatin-associated proteins. For example, FRAP and two-photon photoactivation were used to determine the exchange dynamics of histone H2B in plant stem cells of *Arabidopsis thaliana* roots [20]. Another strategy to follow in vivo histone modifications is based on the generation of genetically-encoded fluorescent modification-specific intracellular antibodies (*mintbodies*) which provides information regarding the kinetic changes of specific histone modifications e.g., after treatment with epi-drugs (chemical compounds targeting epigenetic regulators) such as trichostatin A (TSA), a histone deacetylase inhibitor [21]. More recently, the time-lapse microscopy has been also used to study the effects of chromatin regulators (CRs) on establishing the transcriptional activity of a reporter gene in single mammalian cells [22]. The authors combined the artificial recruitment of CRs to target a reporter locus with time-lapse microscopy to evaluate transitions between active and inactive states of the reporter gene [22]. Regarding nucleosome mapping, the atomic force microscope (AFM) allows studies on the nucleosomes’ positioning, and on the respective histone-DNA binding forces, as well as on the electrical conductivity of DNA [23]. The chromatin conformation capture (3C) techniques, based on high-throughput sequencing, can generate detailed information regarding chromatin geography, namely the “chromatin loops” which may bring distant DNA regions physically close together in the 3D nuclear space [24,25,26,27]. These approaches have been, so far, mainly used in mammalian genomes, enabling the identification of topologically-associating domains (TADs), defined as regions with high internal interactions, but little interaction with neighbouring regions, and lamina-associated domains (LADs), which can influence gene expression [28,29,30]. The TAD domains, as described in animals, may not be present in plants, as shown in *Arabidopsis* by using chromatin conformation capture methodology [31]. This approach enabled to confirm previous cytogenetic observations made in *Arabidopsis*, further making it possible to decipher, with more precision, specific patterns of chromatin interactions [31]. The distal regions of *Arabidopsis* chromosomes have a higher probability to establish interactions with other chromosomes [32]. Furthermore, specific epigenetic marks, typically associated with gene repression, were associated with a high level of chromatin interactions, while other epigenetic marks related to facilitation of transcription were more distant from the interaction domains [31]. These observations led the authors to suggest that domains of active transcription may not be associated to an increased number of interactions [31].

## 2. Chromatin Flexibility in Response to Stress Factors

Genomes can be restructured upon sensing fluctuations of external environmental conditions and this idea was first advanced by McClintock who visualized extensive remodelling of maize chromosomes in response to mutagen exposition [33]. Later, it was proposed a hypothetical model explaining the involvement of chromatin in genome regulation by stress [34]. Under standard conditions, heterochromatic configurations may be involved in transcription silencing of repetitive DNA sequences while under stress factors may alter epigenetic imprints and, subsequently, heterochromatic states, and all together may generate transposon activation, epigenetic remodelling, and novel transcriptional patterns [34]. The activity of chromatin remodelling factors and epigenetic levels can be sensitive to environmental factors, such as temperature, water and nutrient availability, and light conditions [35,36]. For example, when cold stress is imposed to *Arabidopsis*, there is a recruitment of chromatin remodelling factors to flower repressor loci, which is further accompanied by an enrichment of specific histone modification as the methylation of histone 3 at lysine 9 and 27 (H3K9, H3K27), being that these markers are important for the repression of specific flower genes (reviewed in [37]).

The nucleolar chromatin has been also associated with stress responses to DNA damage [38]. In animal cells, the nucleolus has been suggested to act as a cell sensor for stresses [39]. In addition, the nucleolar proteins can suffer extensive repositioning in response to environmental fluctuations. For example, the nucleolin, which is an abundant factor critical for precursor rRNA (pre-rRNA) processing, moves from the nucleolus to the nucleoplasm following heat stress [40]. The plant nucleolus can actually be a good experimental model to study stress-mediated effects on chromatin remodelling, since the rDNA chromatin can adopt at least three different states: inactive and condensed rRNA genes (heterochromatin) corresponding to “knobs” of highly-packed chromatin mainly positioned at the nucleolus periphery and within some nucleoli [41]; active genes in an extended, decondensed conformation; and a potentially-activated state [42]. The equilibrium between these distinct chromatin conformations is dependent on multiple interactions with specific nucleolar DNA binding proteins, histone modifications, such as acetylation and methylation at particular residues, and also on the incorporation of specific histone variants and DNA methylation [43]. Switching between distinct chromatin conformations may occur in response to abiotic stresses; for example, the germination of rice seeds in a salt solution or the exposure of wheat seedlings to heat stress caused a decondensation of rDNA interphase chromatin associated with a decreased volume of heterochromatic perinucleolar rDNA knobs [35]. Still, the mechanisms behind chromatin flexibility are not yet understood.

### 2.1. Methylome Flexibility under Stress

Plant exposure to abiotic stresses has often been linked to alterations of chromatin structure, and also with changes in the DNA methylation level (Table 1). The DNA methylation level, either at specific genes or at the genome-wide level, can be responsive to environmental changes. For example, tobacco plants subjected to stress (e.g., aluminium, salt, cold, or oxidative stress) acquired a lower DNA methylation specifically at a gene coding for a glycerophosphodiesterase-like protein, which is associated to its higher transcription [44,45]. The imposition of cold stress to maize seedlings generated a genome-wide DNA demethylation, particularly in root tissues [46]. Another example is the activation of the transposon *Tam3* in *Antirrhinum majus* which correlated with DNA demethylation events under low temperatures [47,48]. The imposition of cold stress to *Medicago sativa* plants was also associated with transcription activation of specific retrotransposons, which, in this case, did not necessarily relate to DNA demethylation events [49]. In rice, osmotic stress imposition was associated not only with a higher expression of specific genes with a crucial role on proline biosynthesis (P5CS and δ-OAT), but also with a global DNA demethylation [50]. Tobacco plants under water-deficit stress conditions also displayed specific changes of epigenetic marks, particularly the leaf *Asr1* gene, which showed decreased DNA methylation and less of the H3K27me3 mark, which is interpreted as repressive mark, in combination with an increased transcription [51]. Episodes of DNA demethylation have been a common finding in many studies involving the salt stress imposition [52,53], although it is still not clear the mechanism underlying the methylation/demethylation cycle.

The DNA methylation dynamics have been investigated regarding stress adaptation. Salt-tolerant varieties tend to be more successful in adjusting DNA methylation levels under salinity [53]. Similarly, salt stress applied to *Brassica napus* generates demethylation events, and that was more evident in a variety described as salt-tolerant (“Exagone”), as compared with a salt-sensitive one (“Toccata”) [56]. In the horse gram (*Macrotyloma uniflorum*), a highly drought-tolerant legume, distinct genotypes show distinct sensitivities to drought. Specifically, the drought-sensitive genotype showed higher methylation [64]. Barley genotypes with contrasting behaviour under drought also exhibited differential DNA methylation patterns particularly in what concerns a specific drought stress-responsive gene coding for a DNA glycosylase closely related to the cereal DME-family DNA glycosylases (HvDME) [65]. In the Chernobyl area, the exposition of *Pinus silvestris* to ionizing radiation stress caused a genome hypermethylation that was interpreted as a genome protective strategy, likely increasing plant adaptation and survival under the radiation stress [63]. These studies indicate that certain genomic regions may be more prone to differential methylation upon stress imposition/relief eventually corresponding to a stress adaptation process.

Methylome variations are present in natural plant populations and may help individuals to better cope with different environments. For example, within *Laguncularia racemose* (a mangrove species occurring in naturally-contrasting habitats), individuals from salt marshes show higher methylation level than those located on the riverside [66]. Additionally, varieties of *Cannabis sativa* with distinct cold acclimation capacities can show distinct epigenetic variations [67]. In particular, those varieties with more efficient acclimation capacity showed increased methylation levels at the COR gene loci upon deacclimation, suggesting a link between locus-specific methylation and deacclimation [67]. Collectively, these studies illustrate that methylome can be shaped in response to environmental factors allowing protection and/or adaptation.

### 2.2. Histone Modifications Flexibility under Stress

The accessibility to chromatin is regulated by the histone modification landscape, namely by the spatial distribution, particularly along gene promoter regions of specific histone modification marks, e.g., acetylation or methylation [5,68]. These chemical histone modification marks have a transient nature and are highly responsive to distinct environmental stresses (Table 1), thus justifying expression changes in stress-responsive genes [54,55,59,60,69,70,71]. In *Arabidopsis*, the histone deacetylase AtHD2C modulates abscisic acid (ABA)-responsive genes, playing an important role in enhancing plant tolerance to salt and drought [72]. Furthermore, in *Arabidopsis*, the activation of drought-responsive genes (*RD*29A, *RD*29B, *RD*20, *RAP*2.4) was associated with an enrichment of the H3K4me3 and H3K9ac marks at the coding regions during dehydration stress [59]. After stress application, the H3K9 acetylation marks were removed while H3K4me3 were maintained [60]. In rice, the transcriptional induction of specific dehydrin genes under drought was correlated with an increase of the histone modification mark H3K4me3 [54]. Also, rice plants subjected to submergence showed an increase of H3 acetylation marks associated with the induction of specific stress-responsive genes (*ADH1* and *PDC1*) [55]. The cold condition can also generate extensive restructuring of the maize genome. In particular, the heterochromatic chromatin knobs, essentially made of repetitive sequences, become transcriptionally active concomitantly with an increase of H3K9ac, a histone acetylation mark typically associated with improved transcription competence, and a decrease of H3K9me2, a histone demethylation mark related to transcription-repression [62]. Furthermore, these alterations went together with a decrease of DNA methylation level [62]. In *Arabidopsis thaliana*, cold stress triggered an enrichment of the histone mark H3K27me3 at the promoter region of the *COR15A* and *ATGOLS3* genes, also being associated with increased expression of these genes [61]. Treatments of *Arabidopsis* with ABA or salt also triggered various fluctuations in the level of histone modification marks, namely, an enrichment of the marks H3K9K14ac and H3K4me3, normally associated with gene induction and a decrease of the H3K9me2 mark seen as having a repressive role on gene expression regulation [57]. Additionally, in *Arabidopsis*, the *HKT1* gene that is transcriptionally salt stress-induced, suffered a decrease in the H3K27me3 repressive mark [58]. Furthermore, the dynamics of incorporating specific histone variants is also involved in response to environmental changes. In *Arabidopsis*, a specialized histone H1 variant (H1.3) is required for proper stomatal functioning under normal growth conditions and also for adaptive responses to combined light and water deficiency [73]. The histone H2A.Z variant acts as a thermal sensor by inducing a more compactly wrapped DNA, thus decreasing chromatin accessibility to transcription elements [74]. All together, these reports illustrate a link between stress imposition, fluctuations of specific histone modification marks, and the transcriptional regulation of specific stress responsive genes.

The DNA methylation/demethylation cycle intersect, in a combined way, with histone modifications, but the mechanism behind the process is still not well understood. In animals, there are a few reports showing a correlation between higher levels of histone acetylation and DNA demethylation [75,76]. In *Arabidopsis thaliana*, it was shown that active DNA demethylation can be regulated by methyl-CpG-binding domain proteins [77,78]. These findings point to the existence of a very complex network of concerted interactions between chemical modifications of DNA and histones to coordinate genome function.

## 3. Flexibility of Chromatin Structure Induced by Epigenetic Drugs: A Tool to Understand the Role of Epigenetics in Stress Response

The use of chemical compounds targeting epigenetic mechanisms (epigenetic drugs) can contribute to better understanding of the functional implications of chromatin and epigenetic plasticity in gene expression and phenotype regulation. Epigenetic drugs include demethylating substances that inhibit or affect DNA methyltransferase action, and chemical compounds that can affect histone acetylation levels. The drug 5-azacytidine (5-AC) has been widely used in various organisms as a tool to significantly reduce DNA methylation levels [79]. Early work showed that 5-AC was able to induce decondensation of constitutive heterochromatin segments and G-band chromatin on human chromosomes, as well as inducing chromosome fragility and micronucleus formation [80,81]. In wheat-rye hybrid lines, seed germination in 5-AC led to alterations in the 3D physical disposition of parental genomes in interphase nuclei, namely to an increase of intermingling between wheat and rye genomes [82,83]. In the same way, the germination of wheat seeds in 5-AC also caused large-scale decondensation of interphase chromosome territories and of rDNA chromatin [12,35,84]. In rice, the 5-AC treatment was also associated with decondensation of rDNA chromatin, as well as to alterations of centromere disposition in interphase nuclei, pointing to a link between DNA methylation and interphase chromatin organization [35]. General criticisms about using 5-AC for chromatin and epigenetic functional studies relate to its instability in aqueous solution, high toxicity, and pleiotropic and unpredictable effects. Zebularine (1-(β-D-ribofuranosyl)-1,2-dihydropyrimidine-2-one) is another cytidine analogue, originally discovered in the filamentous fungus *Neurospora* [85], which acts in a similar manner as 5-AC, but with the advantage of being more stable and less toxic [86]. Zebularine was reported to reduce methylation in a dose-dependent and transient manner affecting whole genome transcription and causing the dispersion of heterochromatic chromocenters in *Arabidopsis* [86].

Specific mutations affecting chromatin and epigenetic modulators can contribute to the understanding of the impact of deregulated epigenetic backgrounds in genome function and stress response. For example, in mice, a knockout mutation of a methyltransferase (*Dnmt1*) led to incapacity in keeping DNA methylation, which was associated with embryo-lethality, highlighting the crucial role of DNA methylation for the completion of early development [87]. Contrastingly, in the filamentous fungus *Neurospora,* mutants showing DNA hypomethylation only exhibited mild and variable morphological defects [88]. Regarding plants, the DNA methylation pathway can also be affected through specific mutations. For example, *Arabidopsis thaliana* mutants with decreased DNA methylation levels (DDM, for decrease in DNA methylation) did not show dramatic changes on morphological parameters [89]. However, when *ddm1* mutant plants went through successive rounds of self-pollination, there is an increased loss of DNA methylation along with several phenotypic and developmental abnormalities [90]. Furthermore, in the *ddm1 Arabidopsis* mutants, the low level of methylation was associated with higher plant sensitivity to salt stress and to less ability to repair DNA damage after exposure to UV-B stress [91]. Additionally, in *Arabidopsis*, the *drm1drm2* double mutants lacking the de novo methylation did not display significant differences regarding specific phenotypic features in comparison to the wild-type, even after subsequent generations of inbreeding [92]. Regarding rice, a mutation in a DNA methyltransferase (*osdrm2*) affecting the de novo methylation was associated with multiple developmental abnormalities, such as growth retardation, morphological defects on panicles and spikelet, as well as a strong sterility [93]. Furthermore, the seeds of the *osdrm2* rice mutants were slower to germinate than the WT (Dongjin) seeds [53]. The *osdrm2* rice mutant plants were also evaluated regarding performance under stress, the root length, and the biomass, which were not drastically affected by salinity stress [53]. These reports show that it is still difficult to establish direct correlations between DNA methylation levels and phenotypic parameters particularly relevant in stress response.

Histone acetylation levels can be altered by using specific inhibitors of the enzymes controlling acetylation and deacetylation. In the 1970s, it was isolated an antifungal antibiotic, trichostatin A (TSA), from *Streptomyces hygroscopicus* [94]. A clue to understanding the target of the TSA drug was obtained from the analysis of histone modifications by acid urea triton gel electrophoresis, which separates core histone molecules with different acetylation levels. This experiment revealed that, in various mammalian tumour cell lines treated with TSA, the histones were globally hyperacetylated [95]. The use of distinct drugs, such as 5-AC and TSA, separately or simultaneously, can also help to clarify the role of intersecting DNA methylation and histone deacetylation in gene regulation. The transgene locations within the interphase nucleus of wheat transgenic lines, containing multiple transgene integration sites of a reporter gene (GUS), were analysed, since it is during interphase that (trans) gene expression mainly occurs. On metaphase chromosomes, the transgenes were mapped to various single and multiple chromosomal locations. However, during interphase, these copies were brought together and were often seen as a single labelled focus [11]. Furthermore, in some way, there was always some level of transcriptional silencing of these transgenes [12]. The silencing could be reduced, and the transcription level increased, by treatment with 5-azacytidine (5-AC), which reduces cytosine methylation, or with trichostatin-A (TSA), which inhibits histone deacetylases, thus increasing histone acetylation levels, associated with transcriptional activation [12]. These drugs induced changes in epigenetic levels that were associated with the separation of the transgene copies, which were then visualized as strings of dots [12]. In *Arabidopsis*, the use of 5-AC or TSA drugs, separately or together, showed non-synergistic induction of global DNA hypomethylation or histone hyperacetylation, at least for the majority of the responsive genes [96]. In maize, treatments with 5-AC and TSA drugs caused a reduction of the mitotic index, suggesting an involvement of epigenetic changes in triggering mitosis of root tip cells [97]. In tobacco, the 5-AC treatment caused a striking decrease of methylation of the *npt*II transgenes which led to increased levels of NPTII protein [98].

Mutants in whom histone acetyltransferases and histone deacetylases (HDAs) are affected can generate pleiotropic effects, putatively influencing plant responses to stress. For example, a mutation for a histone acetyltransferase (*oshac704*) in rice caused a significant reduction of spikelet fertility in plants grown in no-stress conditions [53]. Interestingly, these *oshac704 rice* mutants had a lower level of global methylation than the WT plants [53]. In *Arabidopsis*, the histone deacetylation mutation (HDA6) was also associated with a delayed-flowering phenotype [99]. These examples illustrate a concerted intersection between distinct epigenetic layers generating multiple, and still unpredictable, pleiotropic effects that may influence stress responses.

## 4. Chromatin and Epigenetic Mechanisms Are Implicated in “Plant Memory” of Past Stress

Plants may have developed an effective way of knowledge transference regarding stress experiences to their offspring, aiming to anticipate better responses of descendants under abnormal situations [100]. Whether plants are effectively able to transmit specific epigenetic marks or chromatin configuration induced by stress related experiences between sequential generations is still under discussion [101]. One of the questions is whether stress-induced epigenetic changes may encode a “memory” of environmental stress being heritable through mitosis, and in some situations through meiosis [102]. In animals, epigenetic marks (specifically methylation) tended to be described as not being transferred to progeny, occurring instead in epigenetic reprogramming and resetting during gametogenesis or embryo pre-implantation [103]. However, a study with embryos of *Caenorhabditis elegans* pointed to the transmissibility of specific epigenetic marks such as the H3K27me mark, which is typically interpreted as having a role in establishing gene repression [104]. In plants, it is not yet clear if such resetting mechanisms can exist during meiosis, but if it can, it should be incomplete, since a few specific epigenetic marks were already reported to be maintained and transmitted to progeny [105,106,107,108]. In fact, in plants, there are several studies showing that stress-induced epigenetic marks in parent plants can be transferred across generations, even when plants are not subjected to stress, suggesting that plants can “remember” certain stress conditions, but the underlying mechanisms’ “memories” are still not well understood [106]. DNA methylation has been referred to as a heritable mark with a role in memorizing gene expression patterns with implications at the phenotypic level [109]. For example, in flax (*Linum usitatissimum*) changes in methylation patterns caused by 5-AC treatments persisted for the lifetime of the treated individuals, and could still be detected five to nine generations later [110]. Additionally, the progeny of tobacco plants infected with tobacco mosaic virus (TMV) showed a DNA hypomethylation status at specific loci which was transgenerationally inherited with enhanced TMV resistance in the offspring [111]. The inheritance of epigenetic states was also reported in rice plants treated with a DNA hypomethylating drug (5-AC) followed by progeny cultivation in the field for >10 years. In this case, the demethylation state of the *Xa21G* gene was stably inherited and, moreover, conferred increased resistance to *Xanthomonas oryzae* [112]. Also in rice, drought-related DNA methylation changes were shown to be transmitted across six generations [113]. The exposure of *Arabidopsis thaliana* plants to stresses, e.g., salt, UVC, cold, heat, and flooding, resulted in higher levels of homologous recombination that persisted for at least four consecutive generations of untreated progenies [114]. In addition, the untreated progeny of *Aradidopsis* plants also displayed an increased global DNA methylation and a higher tolerance to stress, however, in this case, these effects were not reported as being transmitted across generations [115]. *Arabidopsis* primed plants (i.e., plants previously exposed to stress) can be used to make plants more resistant to further stress exposures [108]. Interestingly, the progeny of these plants, when challenged with a pathogen, showed enhanced expression of defence-related genes and enhanced disease resistance when compared to the progeny of non-primed plants [108]. Collectively, these studies indicate a putative role of transgenerational epigenetic inheritance in adaptation to environmental stresses. If, on the one hand, the inheritance of epigenetic marks induced by stress can eventually bring benefits regarding adaptation to environmental challenges, on the other hand, the cumulative effects of chromatin changes may hamper prompt responses to the current environment [116]. These authors identified specific loci and chromatin factors with a role in preventing a massive transgenerational inheritance of epigenetic marks related to stress [116].

## 5. Conclusions

The current and future trends for chromatin and epigenetic studies should depend on the in vivo topography of epigenetic marks in a single cell. In this sense, the chromatin conformation capture (3C) techniques have brought high-throughput approaches to three-dimensional studies of chromatin conformation, identifying transient interactions of interphase chromatin domains within living cells. The functional mechanisms behind chromatin and epigenetic plasticity in response to transcription requirements or stress conditions are still not well understood and the connection between epigenetic factors with phenotype regulation is even less known. Further research needs to be done in crop plants to better clarify the role of epigenetic factors in establishing a set of “stress memories” with the expectation of finding, in the future, epigenetic-based key markers for improved plant adaptation to stress conditions.

## Figures and Tables

**Table 1:** Some examples of chromatin flexibility in relation to abiotic stress responses.

Plant Species	Stress Type	Chromatin Alterations	References
*Oryza sativa*	Salt	↑ rDNA chromatin decondensation	[35]
		↓ Genome-wide DNA methylation	[52,53]
	Water-deficit	↑ H3K4me3 regarding the dehydrin genes	[54]
	Submergence	↑ H3ac regarding the *ADH1* and *PDC1* genes	[55]
	Osmotic	↓ Genome-wide DNA methylation	[50]
*Nicotiana tabacum*	Aluminium; Salt Cold; Oxidative	↓ DNA methylation of the *NtGPDL* gene	[44,45]
*Solanum lycopersicum*	Water-deficit	↓ DNA methylation; ↓ H3K27me3 regarding the Asr1 gene	[51]
*Brassica napus*	Salt	↓ Genome-wide DNA methylation	[56]
*Arabidopsis*	Salt	↑ H3K9ac; ↑ H3K4me3; ↓ H3K9	[57]
		↓ H3K27me3 regarding the *HKT1* gene	[58]
	Drought	↑ H3K4me3; ↑ H3K9ac regarding the *RD29A, RD29B*, *RD20*, *RAP2.4* genes	[59,60]
	Cold	↑ H3K27me3 regarding the *COR15A* and *ATGOLS3* genes	[61]
*Triticum aestivum*	Heat	↑ rDNA chromatin decondensation	[35]
*Zea mays*	Cold	↓ Genome-wide DNA methylation; Nucleosome remodelling at tandem-repeat sequences with a: ↓ DNA methylation;↑ H3K9ac; ↓ H3K9me2	[46,62]
*Antirrhinum majus*	Cold	↓ DNA methylation of the transposon Tam3	[48]
*Pinus silvestris*	Ionizing radiation	↑ Genome-wide DNA methylation	[63]
